# Environmental Enrichment Promotes Antioxidant Effect in the Ventrolateral Medulla and Kidney of Renovascular Hypertensive Rats

**DOI:** 10.5935/abc.20190166

**Published:** 2019-11

**Authors:** Luiz Eduardo Sousa, Iuri Ferrari Del Favero, Frank Silva Bezerra, Ana Beatriz Farias de Souza, Andreia Carvalho Alzamora

**Affiliations:** Universidade Federal de Ouro Preto, Ouro Preto, MG - Brazil

**Keywords:** Renovascular Hypertension, Oxidative Stress, Enviromental Enrichment, Ventrolateral Medulla, Kidney

## Abstract

**Background:**

Arterial hypertension is a precursor to the development of heart and renal failure, furthermore is associated with elevated oxidative markers. Environmental enrichment of rodents increases performance in memory tasks, also appears to exert an antioxidant effect in the hippocampus of normotensive rats.

**Objectives:**

Evaluate the effect of environmental enrichment on oxidative stress in the ventrolateral medulla, heart, and kidneys of renovascular hypertensive rats.

**Methods:**

Forty male Fischer rats (6 weeks old) were divided into four groups: normotensive standard condition (Sham-St), normotensive enriched environment (Sham-EE), hypertensive standard condition (2K1C-St), and hypertensive enriched environment (2K1C-EE). Animals were kept in enriched or standard cages for four weeks after all animals were euthanized. The level of significance was at p < 0.05.

**Results:**

2K1C-St group presented higher mean arterial pressure (mmHg) 147.0 (122.0; 187.0) compared to Sham-St 101.0 (94.0; 109.0) and Sham-EE 106.0 (90.8; 117.8). Ventrolateral medulla from 2K1C-EE had higher superoxide dismutase (SOD) (49.1 ± 7.9 U/mg ptn) and catalase activity (0.8 ± 0.4 U/mg ptn) compared to SOD (24.1 ± 9.8 U/mg ptn) and catalase activity (0.3 ± 0.1 U/mg ptn) in 2K1C-St. 2K1C-EE presented lower lipid oxidation (0.39 ± 0.06 nmol/mg ptn) than 2K1C-St (0.53 ± 0.22 nmol/mg ptn) in ventrolateral medulla. Furthermore, the kidneys of 2K1C-EE (11.9 ± 2.3 U/mg ptn) animals presented higher superoxide-dismutase activity than those of 2K1C-St animals (9.1 ± 2.3 U/mg ptn).

**Conclusion:**

Environmental enrichment induced an antioxidant effect in the ventrolateral medulla and kidneys that contributes to reducing oxidative damage among hypertensive rats.

## Introduction

Arterial hypertension is a key precursor to the development of stroke, renal failure, heart failure, endothelial dysfunction and cardiovascular disease. Oxidative stress has been implicated in the pathogenesis of arterial hypertension and other cardiovascular diseases.^1,2^ Imbalance from excessive oxidative compounds coupled with lowered antioxidant defences induces cellular damage.^1^ In hypertensive rats, oxidative stress is linked to heart, kidney, and brain dysfunction.^3-5^ Furthermore, reactive oxygen species [ROS] in the ventrolateral medulla are major contributors to sympathetic hyperactivity and arterial hypertension.^4,6^

The brainstem contains complex structures that control blood pressure and cardiovascular reflex.^7,8^ These regions include the caudal pressor area, caudal ventrolateral medulla (CVLM), and medullocervical pressor area.^7,9^ Inhibition and activation of these nuclei cause depressor and pressor responses, respectively. The rostral ventrolateral medulla (RVLM) is an important area of basal sympathetic tone, under regulation by CVLM neurons via tonic and phasic inhibition.^9^ Rat studies show that hypertension is associated with lower antioxidant defences and elevated oxidative markers in both CVLM and RVLM.^3,4,6,10^

Besides the central nervous system, heart and kidneys are also involved in cardiovascular control.^5^ When arterial hypertension develops in renovascular hypertensive rats, both organs experience redox imbalance, along with the increased inflammatory-cell count, lipid peroxidation, and reduced antioxidant defences.^5^

A sedentary lifestyle is a risk factor for cardiovascular disease, including arterial hypertension.^11^ Physical exercise improves antioxidant defence in the ventrolateral medulla, thus reducing oxidative damage and hypertension.^3,12,13^ One way to raise motor activity in laboratory animals is with environmental enrichment. Animals in enriched housing experience neuroanatomical, chemical, and behavioural changes that heighten memory, motor function, sensorial and cognitive activity.^14-16^ In turn, neuroplasticity is increased while neurodegenerative disorders and cognitive decline are mitigated. Environmental enrichment of rodents reliably increases performance in learning and memory tasks, and improves overall health and well-being, compared with standardly housed conspecifics.^14,17,18^ Such enrichment typically involves introducing stimuli such as toys, tubes, balls, igloos, running wheels, or any items that provoke exploration, social interaction, sensorial and motor activity.

Non-motor environmental enrichment (e.g. without wheel running) also appears to exert an antioxidant effect in the hippocampus and cerebral cortex of normotensive rats.^15,16^ Thus, environmental enrichment may reduce oxidative stress in the ventrolateral medulla and contribute to hypertension control. Yet despite noted effects on cognitive activity, no consistent data are available regarding exactly how enrichment influences oxidative stress in the ventrolateral medulla of hypertensive animals. The aim of this study is to evaluate the effect of environmental enrichment on oxidative stress in the ventrolateral medulla, heart, and kidneys of renovascular hypertensive rats.

## Methods

### Animal experiments

All animal experiments in the study were approved by the Ethics Committee on Animal Use of the Universidade Federal de Ouro Preto by protocol number 2016/24. Subjects were forty adult Fischer male rats (6 weeks old; 140-170 g). Renovascular hypertension was induced as previously described.^19^ Briefly, animals were anaesthetized with 50 mg/kg of ketamine and 10 mg/kg of xylazine (i.p.). Next, a silver clip (2K1C; inner diameter, 0.2 mm) was placed around the left renal artery through a midline incision. Normotensive groups (Sham) were subjected to similar procedures but without clips. Four groups were formed: standardly housed hypertensive (2K1C-St) (n = 12), enriched hypertensive (2K1C-EE) (n = 8), standardly housed normotensive (Sham-St) (n = 7), and enriched normotensive (Sham-EE) (n = 13). The sample size was defined by convenience.

### Environmental enrichment and standard condition protocol

**Figure 1:**
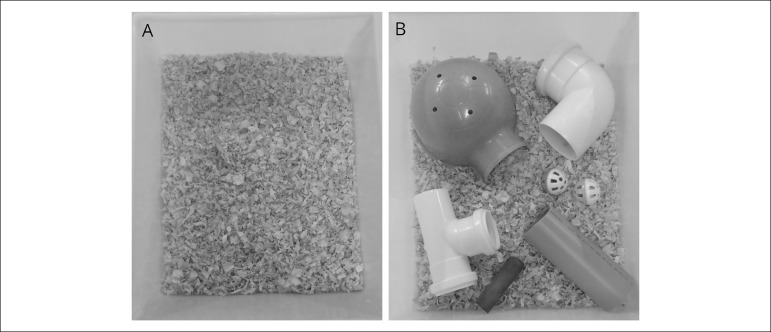
Representation of the environments that rats were exposed (A) standard cage (B) enriched cage.

Immediately post-surgery, rats were housed in enriched (EE) or standard (St) cages (Figure 1). The former contained six different objects (plastic coloured balls, igloo, wooden chew sticks, curved tubes, straight tubes); cages were organised with four rats in each cage. Eight different objects combinations were used, with one weekly change to encourage exploration. The control environment consisted of standard cages (St) identical to those used for housing subjects during non-experimental periods. All groups were kept in the same room, on different racks, to control for lighting (12-12 h light-dark cycle), temperature (24 ± 1ºC), humidity, and ambient noise. Rat chow and tap water were provided ad libitum. The enrichment procedure lasted for four weeks. At the end of that time, cardiovascular measurements were performed.

### Cardiovascular measurements

Within 2 days of experiment end, rats were anaesthetised with urethane (1.2 g/kg body weight, ip; Sigma-Aldrich, USA). Polyethylene catheters (PE-10 connected to PE-50, Clay Adams, Parsippany, NJ, USA) filled with heparinized saline (400 IU/mL) were inserted into the right femoral artery for measuring pulsatile arterial pressure. Blood pressure was recorded at 1000 Hz sampling rate and 20 mV range digitizing window. Heart rate and mean arterial pressure (MAP) were derived on-line from the pulsatile arterial pressure signal with LabChart for Windows software (ADInstruments Pty Ltd, Australia). Experimental protocols were carried out in anaesthetized rats. After cardiovascular measurements, rats were decapitated and the left ventricle was perfused with saline solution to remove blood from tissues. Brain, hearts, kidneys and the ventrolateral medulla was dissected, and all samples were frozen in liquid nitrogen before storage at -80ºC.

### Antioxidant defences and oxidative stress biomarkers

Superoxide dismutase (SOD) activity was evaluated via the enzyme’s capacity to inhibit pyrogallol autoxidation (absorbance of 570 nm).^20^ Catalase activity was determined through the speed of H_2_O_2_ reduction (absorbance of 240 nm).^21^ Carbonyl protein determination methods (absorbance of 370 nm) were based on a previous publication.^22,23^ Lipid peroxidation was evaluated with the thiobarbituric acid-reactive substances (TBARS) assay (absorbance of 535 nm).^24^ Total protein was quantified with the Bradford method.^25^

### Statistical analysis

Results are reported as means ± SD or median, minimum and maximum value. Normality was checked using Kolmogorov-Smirnov. Data were then analysed with one-way ANOVA test, followed by a post-hoc Newman-Keuls test or Kruskal-Wallis test, followed by Dunn’s post-test. Statistical analyses were performed in GraphPad Prism. Significance was set at p < 0.05.

## Results

### Effects of environmental enrichment on mean arterial pressure

**Figure 2:**
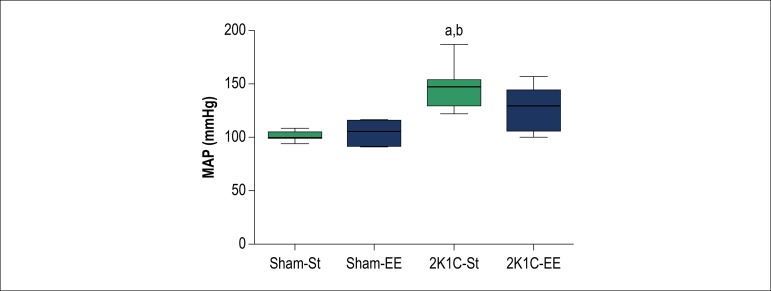
Baseline levels of mean arterial blood pressure (MAP, mmHg) in hypertensive (2K1C) and normotensive rats (Sham) subjected to environmental enrichment (EE) and standard condition (St). Direct measurements of MAP were performed on the 4th weeks after 2K1C surgery. Letters (above the bars) indicate significant differences. (a) The difference in relation to the Sham-St; (b) Difference in relation to the Sham-EE analysed by Kruskal-Wallis test, followed by Dunn’s post-test a (p < 0.05) (n = 7 in each group). Data are expressed as median, minimum and maximum value.

To evaluate if environmental enrichment reduces blood pressure in hypertensive rats, we compared MAP across our four experimental groups. The 2K1C-St group had significantly higher MAP 147.0 (122.0; 187.0) mmHg, p < 0.05, than Sham-St 101.0 (94.0; 109.0) mmHg and Sham-EE 106.0 (90.8; 117.8) mmHg. The four groups did not differ significantly in heart rate (Figure 2).

### Effects of environmental enrichment on oxidative stress marker in the ventrolateral medulla

Superoxide dismutase activity in the ventrolateral medulla was higher for 2K1C-EE than for 2K1C-St. Additionally, catalase activity was higher in 2K1C-EE than in all other groups. We observed increased TBARS value in 2K1C-St compared with Sham groups, but reduced activity in 2K1C-EE compared with 2K1C-St (Table 1).

**Table 1:** Effects of environmental enrichment or standard conditions on the activity of antioxidant enzymes and biomarkers of oxidative damage in the ventrolateral medulla

	SOD (U/mg ptn)	Catalase (U/mg ptn)	Carbonyl protein (nmol/mg ptn)	TBARS (nmol/mg ptn)
Sham-St (n = 7)	32.9 ± 7.1	0.3 ± 0.04	22.1 ± 12.1	0.29 ± 0.12
Sham-EE (n = 13)	41.8 ± 11	0.4 ± 0.2	28.6 ± 11.9	0.36 ± 0.1
2K1C-St (n = 12)	24.1 ± 9.8	0.3 ± 0.1	44.6 ± 15.1	0.53 ± 0.22^a,b^
2K1C-EE (n = 8)	49.1 ± 7.9^c^	0.8 ± 0.4^a,b,c^	57.1 ± 12.5^a,b^	0.39 ± 0.06^c^

a represents a significant difference between groups when compared to Sham-St;

b represents a significant difference between groups when compared to Sham-EE;

c represents a significant difference between groups when compared to 2K1C-St.

Analysed by one-way ANOVA, followed by Newman Keuls post-test (p < 0.05). Data are expressed as the mean ± SD (n = 7-13 in each group). Sham-St: normotensive group exposed to standard condition; Sham-EE: normotensive group exposed to environmental enrichment; 2K1C-St: hypertensive group exposed to standard condition; 2K1C-EE: hypertensive group exposed to environmental enrichment. SOD: superoxide dismutase; TBARS: thiobarbituric acid-reactive substances.

### Effects of environmental enrichment on oxidative stress marker in the left ventricle

Overall, TBARS concentration was higher in both hypertensive groups than in Sham groups. Catalase activity was lower in 2K1C-St than in Sham-EE. Carbonyl protein and SOD were similar across all experimental groups (Table 2).

**Table 2:** Effects of environmental enrichment or standard conditions on the activity of antioxidant enzymes and biomarkers of oxidative damage in the left ventricle

	SOD (U/mg ptn)	Catalase (U/mg ptn)	Carbonyl protein (nmol/mg ptn)	TBARS (nmol/mg ptn)
Sham-St (n = 7)	12.0 ± 2.9	0.3 ± 0.1	30.1 ± 18.1	0.3 ± 0.17
Sham-EE (n = 13)	12.6 ± 3.8	0.4 ± 0.14	30.1 ± 10.7	0.2 ± 0.14
2K1C-St (n = 12)	9.9 ± 1.4	0.2 ± 0.09^b^	37.2 ± 12.8	0.8 ± 0.07^a,b^
2K1C-EE (n = 8)	12.1 ± 4.2	0.3 ± 0.11	48.2 ± 19	1.1 ± 0.2^a,b^

a represents a significant difference between groups when compared to Sham-St;

b represents a significant difference between groups when compared to Sham-EE.

Analysed by one-way ANOVA, followed by Newman Keuls post-test (p < 0.05). Data are expressed as the mean ± SD (n = 7-13 in each group). Sham-St: normotensive group exposed to standard condition; Sham-EE: normotensive group exposed to environmental enrichment; 2K1C-St: hypertensive group exposed to standard condition; 2K1C-EE: hypertensive group exposed to environmental enrichment. SOD: superoxide dismutase; TBARS: thiobarbituric acid-reactive substances.

### Effects of environmental enrichment on oxidative stress marker in the right kidney

We observed increased SOD activity in environmentally enriched groups (2K1C-EE, Sham-EE) compared with standard groups (2K1C-St, Sham-St). The 2K1C-EE group had higher carbonyl protein and lower TBARS concentration than the 2K1C-St group (Table 3).

**Table 3:** Effects of environmental enrichment or standard conditions on the activity of antioxidant enzymes and biomarkers of oxidative damage in the right kidney

	SOD (U/mg ptn)	Catalase (U/mg ptn)	Carbonyl protein (nmol/mg ptn)	TBARS (nM/mg ptn)
Sham-St (n = 7)	8.1 ± 2.5	1.8 ± 0.7	5.6 ± 2.1	0.3 ± 0.13
Sham-EE (n = 13)	12 ± 3.5^a^	1.9 ± 0.8	4.4 ± 1.9	0.3 ± 0.09
2K1C-St (n = 12)	9.1 ± 2.3	1.5 ± 0.34	3.1 ± 2	0.4 ± 0.18
2K1C-EE (n = 8)	11.9 ± 2.3^c^	2.4 ± 1.1	7.2 ± 2.9^c^	0.1 ± 0.07^c^

a represents a significant difference between groups when compared to Sham-St;

c represents a significant difference between groups when compared to 2K1C-St.

Analysed by one-way ANOVA, followed by Newman Keuls post-test (p < 0.05). Data are expressed as the mean ± SD (n = 7-13 in each group). Sham-St: normotensive group exposed to standard condition; Sham-EE: normotensive group exposed to environmental enrichment; 2K1C-St: hypertensive group exposed to standard condition; 2K1C-EE: hypertensive group exposed to environmental enrichment. SOD: superoxide dismutase; TBARS: thiobarbituric acid-reactive substances.

### Effects of environmental enrichment on oxidative stress marker in the left kidney

Furthermore, SOD activity was higher among 2K1C-EE than 2K1C-St animals. Catalase activity was lower in hypertensive groups (2K1C-St and 2K1C-EE) than Sham groups (Sham-St and Sham-EE). Carbonyl protein activity and TBARS concentration did not differ significantly across experimental groups (Table 4).

**Table 4:** Effects of environmental enrichment or standard conditions on the activity of enzymes and biomarkers of oxidative damage in the left kidney

	SOD (U/mg ptn)	Catalase (U/mg ptn)	Carbonyl protein (nmol/mg ptn)	TBARS (nM/mg ptn)
Sham-St (n = 7)	14.1 ± 5.9	1.4 ± 0.5	24.9 ± 8.8	0.5 ± 0.19
Sham-EE (n = 13)	17.4 ± 8.7	1.5 ± 0.8	31.9 ± 11.9	0.4 ± 0.2
2K1C-St (n = 12)	11.3 ± 2.2	0.1 ± 0.06^a,b^	25.9 ± 9.3	0.7 ± 0.3
2K1C-EE (n = 8)	23.5 ± 6.2^c^	0.4 ± 0.2^a,b^	30.1 ± 8.1	0.4 ± 0.19

a represents a significant difference between groups when compared to Sham-St;

b represents a significant difference between groups when compared to Sham-EE;

c represents a significant difference between groups when compared to 2K1C-St.

Analysed by one-way ANOVA, followed by Newman Keuls post-test (p < 0.05). Data are expressed as the mean ± SD (n = 7-13 in each group). Sham-St: normotensive group exposed to standard condition; Sham-EE: normotensive group exposed to environmental enrichment; 2K1C-St: hypertensive group exposed to standard condition; 2K1C-EE: hypertensive group exposed to environmental enrichment. SOD: superoxide dismutase; TBARS: thiobarbituric acid-reactive substances.

## Discussion

As found in other studies, standardly housed hypertensive rats exhibited MAP values different than standardly housed normotensive controls. Importantly, this study is the first to demonstrate similar MAP values between normotensive and enriched hypertensive rats. This outcome is likely due to the antioxidant effect of enrichment on the ventrolateral medulla and kidneys. The finding that hypertensive rats (2K1C-St) showed higher values of MAP than control animals (Sham-St) is consistent with obtained by other studies.^3,5^

The two-kidney, one-clip (2k1c) Goldblatt hypertensive model is characterized by hyperactivity of the renin-angiotensin system, especially by angiotensin II. Angiotensin II has actions at AT_1_ receptors to elevate blood pressure via numerous mechanisms including vasoconstriction, oxidative stress and increases sympathetic neural discharge. In addition to this classical circulating system, components of the renin-angiotensin system are found locally in the brain. Angiotensin II AT_1_ receptors are abundant in the autonomic nervous system and regulatory brain regions, to influence neurotransmission and blood pressure. The actions of angiotensin II in the ventrolateral medulla contribute to sympathoexcitation and hypertension in animals, in part by stimulating oxidative stress.^26^

The present study showed that the enrichment was effective in making MAP of 2K1C-EE rats similar to that of sham rats. Additionally, the enrichment restored the ROS components in ventrolateral medulla, kidney and left ventricle. Previous studies have showed a hyperactivity of Ang II in both RVLM^27,28^ and CVLM^9,29^ areas of ventrolateral medulla on 2K1C rats. In addition, Ang II has been correlated with ROS in these brain areas.^30^ On the other hand, Ang II is a strong inductor of ROS generation in the ventricle and kidney.^31,32^

Antioxidant compounds are essential to protecting against cardiovascular and nervous systems. Here, the ventrolateral medulla of 2K1C-EE rats had higher SOD and catalase activity in relation to 2K1C-St rats. Thus, environmental enrichment appears to protect against oxidative stress through increasing antioxidant defence, thereby reducing MAP in hypertensive rats. Study shows that increase of antioxidants defences in the ventrolateral medulla reduces the MAP of hypertensive rats.^6^ Likewise, previous research found that spontaneously hypertensive rats experienced a decrease in RVLM SOD activity, but also decreased MAP and sympathetic nerve activity when SOD was overexpressed.^33^

Carbonyl protein concentration was higher in the ventrolateral medulla of 2K1C-EE rats compared with control groups (Sham-St and Sham-EE). This surprising result suggests that environmental enrichment was either detrimental to hypertensive rats, or the ventrolateral medulla is simply less capable of dealing with protein oxidation, even under advantageous conditions. Similarly, a previous study found that environmental enrichment does not reduce protein oxidation in the cortex of male normotensive rats.^16^ However, we also demonstrated that enrichment lowered the TBARS concentration in the ventrolateral medulla of 2K1C rats, indicating that endogenous antioxidants were successfully triggered to neutralise lipid peroxidation. These results corroborate previous research in normotensive rats.^16^ Oxidative stress in the ventrolateral medulla increases sympathetic nerve activity, suggesting that this brain region is important to modulating ROS-induced elevation.^28^

Overall, environmental enrichment appears to exert a neuroprotective effect through ameliorating oxidative stress, the mechanism of neuroprotection has not been established. Oxidative stress is involved in the pathogenesis of acute and chronic neurological diseases. This is because the nervous tissue is uniquely sensitive to oxidative stress, due to the great quantity of sources of ROS.^1^ The synthesis of trophic factors has been suggested to play a role in mediating the neuroprotective effects of enrichment. Environmental enrichment has been found to cause increased brain-derived neurotrophic factor (BDNF) in the glial cell, striatum and substantia nigra.^34^ Thus, we suggest that environmental enrichment is not only relevant to brain aging, dementia and neurodegenerative diseases, but also to other neurological disorders and cardiovascular diseases.

Oxidative stress in the heart is an essential causal agent of hypertension, activating intracellular signalling that induces heart dysfunction through apoptosis or cell overgrowth.^10,35^ We found higher left-ventricle TBARS levels and lower catalase activity in 2K1C-St rats than in Sham (St and EE) rats. Consistent with our results, sedentary 2K1C rats had higher TBARS levels in the left ventricle. In general, renovascular hypertension is expected to increase relative heart weights, cardiomyocyte diameter, myocardial inflammatory cell count, and collagen deposition in the left ventricle.^5^

Environmental enrichment did not seem to alter MAP, TBARS, and overall antioxidant defence in the left ventricle of hypertensive rats. Environmental enrichment did not seem to alter MAP, TBARS, and overall antioxidant defence in the left ventricle of hypertensive rats.^36,37^ We hypothesize that environmental enrichment should include physical activity (e.g. wheel running) for antioxidant effects to be observable in the heart of hypertensive rats. Multiple studies examining hypertension and cardiovascular diseases have illustrated that physical activity confers antioxidant effects in the heart.^5,38,39^

We also observed elevated SOD activity in both kidneys of environmentally enriched hypertensive and normotensive rats. Additionally, left-kidney catalase activity decreased in hypertensive rats (2K1C-St and 2K1C-EE) compared with control, probably because it is the clipped kidney. These data suggest that oxidative stress increased in the kidneys of hypertensive animals.^5^ Activation of the renin-angiotensin-aldosterone system causes ROS to increase in the kidney via multiple pathways, including elevation of NADPH oxidase, mitochondrial dysfunction, decreased NO availability, and decreased antioxidant enzymes. Accordingly, high angiotensin II-related ROS generation is implicated in hypertension development among several animal models, including 2K1C rats.^40,41^

Catalase and SOD activity is reduced in clipped and nonclipped renal cortices of 2K1C pigs. Relatedly, 8-isoprostane PGF_2_ and malondialdehyde excretion are elevated in the kidneys of 2K1C rats.^40,42^ Increased ROS production, induced by angiotensin II infusion, in kidneys can initiate hypertension. SOD protects the kidney against stress by attenuating renal p22^phox^ expression, NADPH oxidase activation.^43^ Moreover, SOD overexpression of in the kidney mitigates oxidative stress and hypertension. Adenoviral gene transfer of human SOD-3 in spontaneously hypertensive rats increased SOD-3 expression in kidneys and blood vessels, thereby reducing vascular O_2_^•-^ while improving endothelial function and vascular reactivity.^44^

Modifications to the standard caging to create enriched environments that allow improved motor activity, visual activity, exploration and social interactions have been associated with significant behavioural changes and improvements in overall health and well-being.^45^ Overall health and well-being are related to lower levels of cortisol and blood pressure; however few studies show its effects on redox system in the hypertensive animals.^46^

## Conclusion

The physiological effects of environmental enrichment are varied, but improved animal welfare and cognitive properties appear to be clear benefits.^15,47^ Our study presents limitations since the laboratory environment places several physical and operational restrictions on the methods and the extent of the enrichment offered to the research mice. These limitations stimulate our ability to use different methods and produce maximum environmental enrichment. Despite the limitations collectively, our results suggested that environmental enrichment conferred antioxidant effects on the ventrolateral medulla and kidneys, probably contributing to MAP reduction and minimizing oxidative damage in renovascular hypertensive rats. We suppose that the enrichment may have made MAP of 2K1C-EE rats similar to SHAM rats by reducing levels of oxidative stress in the ventrolateral medulla, kidneys and heart probably because it reduced Ang II levels in those tissues. However, the effect of environmental enrichment on the renin-angiotensin system is still unknown. No studies were found that show the relationship between enriched environment and the renin-angiotensin system in hypertensive rats. Certainly, new studies should be conducted to clarify this issue.
